# Sodium and potassium urinary excretion and dietary intake: a cross-sectional analysis in adolescents

**DOI:** 10.3402/fnr.v60.29442

**Published:** 2016-04-11

**Authors:** Carla Gonçalves, Sandra Abreu, Patrícia Padrão, Olívia Pinho, Pedro Graça, João Breda, Rute Santos, Pedro Moreira

**Affiliations:** 1 Faculty of Nutrition and Food Sciences, University of Porto, Porto, Portugal; 2 Research Centre in Physical Activity, Health and Leisure, Faculty of Sport, University of Porto, Porto, Portugal; 3 Institute of Public Health, University of Porto (ISPUP), EPIUnit-Epidemiology Research Unit, Porto, Portugal; 4 LAQV/REQUIMTE, University of Porto, Porto, Portugal; 5 Directorate General of Health, Lisbon, Portugal; 6 Division of Noncommunicable Diseases and Health through the Life-Course, World Health Organization Regional Office for Europe, UN City, Denmark; 7 Maia University Institute, Maia, Portugal; 8 Early Start Research Institute, School of Education, Faculty of Social Sciences, University of Wollongong, Australia

**Keywords:** urinary sodium, urinary potassium, intake, adolescents, salt

## Abstract

**Background:**

Hypertension is the leading cause for heart disease and stroke, for mortality and morbidity worldwide, and a high sodium-to-potassium intake ratio is considered a stronger risk factor for hypertension than sodium alone.

**Objective:**

This study aims to evaluate sodium and potassium urinary excretion, and assess the food sources of these nutrients in a sample of Portuguese adolescents.

**Design:**

A cross-sectional study with a sample of 250 Portuguese adolescents. Sodium and potassium excretion were measured by one 24-h urinary collection, and the coefficient of creatinine was used to validate completeness of urine collections. Dietary sources of sodium and potassium were assessed using a 24-h dietary recall.

**Results:**

Valid urine collections were provided by 200 adolescents (118 girls) with a median age of 14.0 in both sexes (*p*=0.295). Regarding sodium, the mean urinary excretion was 3,725 mg/day in boys and 3,062 mg/day in girls (*p<*0.01), and 9.8% of boys and 22% of girls met the World Health Organization (WHO) recommendations for sodium intake. Concerning potassium, the mean urinary excretion was 2,237 mg/day in boys and 1,904 mg/day in girls (*p<*0.01), and 6.1% of boys and 1.7% of girls met the WHO recommendations for potassium intake. Major dietary sources for sodium intake were cereal and cereal products (41%), meat products (16%), and milk and milk products (11%); and for potassium intake, main sources were milk and milk products (21%), meat products (17%), and vegetables (15%).

**Conclusions:**

Adolescents had a high-sodium and low-potassium diet, well above the WHO recommendations. Health promotion interventions are needed in order to decrease sodium and increase potassium intake.

Evidence demonstrates that high sodium intake increases blood pressure and has an impact in endothelial dysfunction, cardiovascular function and structure, kidney disease, and cardiovascular morbidity and mortality (1, 2). Hypertension is recognized as a primary risk factor of heart disease and stroke, both leading causes of death worldwide (3). Recent data regarding sodium intake show that populations around the world are consuming far more sodium than is physiologically necessary (4). In response, the World Health Organization (WHO) considers the reduction of sodium intake to be a priority concern (5), and WHO member states have agreed to work toward a global reduction of 30% until 2025 (6).

At the same time, interest in potassium intake has grown, mainly because potassium attenuates sodium's negative effects, by reducing stroke rates and cardiovascular risk (7) and increasing urinary sodium excretion (8). Furthermore, low dietary intakes of potassium potentiate the sodium sensitivity of blood pressure (9) and the risk of hypertension, while the relationship between sodium and blood pressure strengthens if the urinary sodium-to-potassium ratio (Na^+^/K^+^) is considered instead of only sodium excretion rate (10). High Na^+^/K^+^ ratio intake is considered to be a stronger risk factor of hypertension and cardiovascular disease than each of these nutrients alone (11, 12), and the benefits of a higher intake of potassium are particularly important when sodium intake is high (13).

Data from around the world suggest that the average potassium consumption in the populations of many countries is less than the 2,730–3,120 mg/day, the reference value recommended by the 2002 Joint WHO/Food and Agriculture Organization expert consultation (14).

The WHO recommends a maximum sodium intake of 2,000 mg/day (15) for children and a potassium intake of at least 3,510 mg/day, which should be adjusted downward for children based on their energy requirements (16).

In Portugal, cerebrovascular and cardiovascular diseases are the major causes of death (17), and nearly 42% of the adult population has hypertension (18). In the adult population, the latest data indicate an average consumption of 4,200 mg sodium/day and 2,900 mg potassium/day (18), whereas data regarding the youngest population remain scarce. To the best of our knowledge, no study has yet characterized sodium and potassium intake in Portuguese adolescents using the method of 24-h urine collection (19). This method is considered the ‘gold standard’ for assessing the distribution and average intake of sodium in a representative population (20, 21).

Accurate estimates of sodium and potassium intake are essential for monitoring the effectiveness of current actions to reduce sodium intake and to improve efforts to increase potassium consumption.

In this study, we thus aimed to 1) describe dietary intakes of sodium and potassium and Na^+^/K^+^ ratio; 2) assess their compliance with sodium and potassium intake guidelines; and 3) investigate the main food sources of total dietary sodium and potassium intake among Portuguese adolescents.

## Methods

### Study design

Data for the present cross-sectional study came from a 3-year follow up study with Portuguese children and adolescents, the Longitudinal Analysis of Biomarkers and Environmental Determinants of Physical activity (LabMed Physical Activity Study). The LabMed Physical Activity Study aimed to evaluate independent and combined associations of dietary intake, physical activity, and sedentary behavior on fitness levels and other factors in 12- to 18-year-old children and adolescents.

From the participating schools all students enrolled in the seventh and tenth grade in 2011 scholar year were invited to participate in the study (*n*=1,678). The sample for this analysis consisted of a convenience subgroup of LabMed Physical Activity Study aged 13 to 18 years (*n*=398) that was invited to perform 24-h urine collection and 250 subjects (63%) voluntarily agreed to perform 24-h urine collections. Urine collections were performed between 2012 and 2014. After a validation control of 24-h urine collection (described below), 50 urine collections were rejected (20%). Thus, the final sample consisted of 200 adolescents (82 boys) with both valid urine collection and corresponding dietary recall.

This study was conducted according to the guidelines laid down in the Declaration of Helsinki and all procedures were approved by the Ethical Commission of University of Porto. Written informed consent was obtained from all subjects and caregivers.

### 24-h Urine collection

Participants were asked to complete a 24-h urine collection. Participants and caregivers received oral and written instructions on how to collect complete 24-h urine samples. They were instructed to discard the first morning void and to collect all urine over the following 24-h, including the first void on the next morning, and the time of the start and finish collection was recorded in a questionnaire. Considering the participant's comfort and for feasibility purposes, urine collections were held on Sundays and, during the collection period, subjects were asked to store the collected urine in a cool place. Urine samples were sent to a certified laboratory to be analyzed for urinary creatinine (mg/day) (Jaffé reaction, Siemens Advia 1650), urinary sodium (mEq/day), and urinary potassium (mEq/day) (indirect ion-selective electrodes methodology, Siemens Advia 1800). The maximum coefficient of variation of laboratorial methods was 0.71% for sodium, 1.2% for potassium, and 4.55% for creatinine. Sodium and potassium excretion was reported in mEq/day; however, for comparative purposes, it was converted to mg/day by using their molecular weight. Na^+^/K^+^ ratio was also calculated as sodium (mg/day) divided by potassium (mg/day).

For 24-h urine collection validation, quality control was used calculating 24-h urinary creatinine excretion in relation to body weight according to age group (22), and valid collections were considered when creatinine was between 0.1–0.245 and 0.117–0.294 mmol/kg/day for boys at 9–13 years and 14–18 years, respectively; and between 0.117–0.244 and 0.129–0.238 mmol/kg/day for girls at 9–13 years and 14–18 years, respectively. If the urine collection was incomplete (*n*=50) or if the subjects took medication on the day of collection (*n*=2), the urine specimen was rejected and not considered for analysis. The subjects who reported taking medication on the day of collection agreed to collect 24-h urine again.

### Dietary record

A 24-h dietary recall refers to the day urine was collected by trained interviewers using a photographic book and household measures to quantify portion sizes (23). Energy and nutritional intake were estimated using an adapted Portuguese version of the nutritional analysis software Food Processor Plus (ESHA Research Inc., Salem, OR, USA). The nutrient content of local food was taken from standard nutrient tables (24), whereas the content of commercial food (e.g. pizza and ready-to-eat-food) was derived from labeled ingredients and nutrients.

The food codes used were categorized into 13 major food groups (25): 1) cereal and cereal products (bread; breakfast cereals; biscuits, cakes, puddings, scones, and doughnuts; pasta, rice, and other cereal-based products); 2) meat products (chicken and turkey dishes; sausages; bacon and ham; red meats); 3) milk and milk products (milks, yogurts, and cheeses); 4) vegetable and potato products (potato products); 5) soups and sauces (vegetable soups and sauces); 6) fruits; 7) fish and seafood dishes (cod fish); 8) oils and fats; 9) egg and egg dishes; 10) sugars, preserves, and confectionery; 11) fast food (pizza; sandwiches, burgers, filled wraps; salted snacks and fried snacks; fried potatoes); 12) beverages (soft drinks; water; hot beverages); 13) other foods (beans, pulses, canned fruit, and pickles). The contribution of these 13 food groups to sodium and potassium intake was calculated. An additional analysis was performed to evaluate the contribution of each food group to total sodium and potassium intake only in participants who ingested foods included in that food group.

For validation of records and to check for underreporting, the ratio of reported energy intake (EI) and estimated basal metabolic rate (BMR) according to Schofield (26) was used taking into account age, sex, body weight, and height. Using the formulas proposed by Goldberg et al. (27), we calculated individual specific cutoffs with the following modifications: using the new estimates of the physical activity level assuming light physical activity given for the different age/sex groups of adolescents (28), using coefficients of variation for EI (23%) (29), and the use one recorded day.

Therefore, records with EI:BMR ratios below the cutoff values, depending on the subject's age and sex, were not considered a plausible measurement of the actual one day EI in our further analysis and were excluded (*n*=22, 11%).

### Anthropometric measures

For weight and height measurements, we used a digital scale (Tanita Inner Scan BC 532, Tokyo, Japan) and a portable stadiometer (Seca 213, Hamburg, Germany), respectively. All measurements were performed with participants in light clothing, without shoes, and according to standard procedures (30).

According to body mass index (BMI)-for-age z-scores from the WHO, participants were classified as thin (<−2 SD), normal weight, overweight (>+1 SD), or obese (>+2 SD) (31). The classification was performed using WHO AnthroPlus Software (32).

### Socioeconomic status

As an indicator of the socioeconomic status of the household, the Family Affluence Scale (FAS) was used (33). The FAS is a four-item questionnaire that helps students report their family income objectively: it evaluates the sum of scores regarding whether the family owns a car, whether the student has his or her own bedroom, the number of family vacations during the past 12 months, and the number of computers the family owns. The final score ranges from 0 to 9 points, with higher scores indicating higher socioeconomic status.

### Statistical analysis

All statistical analyses were performed using SPSS 21.0 Inc. A *p*-value <0.05 was considered to indicate statistical significance. The Kolmogorov–Smirnov test was used to assess the assumption of normality. Independent sample *t*-test or Mann–Whitney U test were performed to compare continuous variables, and the Chi-Square test was used for categorical variables. Dietary sources of sodium and potassium were reported through the 13 food and beverages categories. A proportion to total food sodium or potassium was used to report results as mean percentage contribution of each category. Nutrients and food intake were also energy-adjusted according to residual method (34), and adjusted values were used to evaluate the differences between sexes. Spearman's correlation coefficient was used to identify and test the strength of a relationship between urinary sodium with dietary sodium and urinary potassium with dietary potassium.

## Results

Descriptive characteristics of the sample are presented in Table 1. Adolescents were on average 14 years old and most exhibited normal BMI.

**Table 1:** Sample characteristics of the study sample (13–18 years) (*n* = 200)

	Boys	Girls	*p*
Age (years)^a^,^b^	14.7±1.3	14.9±1.4	0.30
Weight (kg)^a^,^b^	60.2±12.2	55.1±9.3	0.01
Height (m)^a^,^b^	1.7±0.1	1.6±0.1	<0.01
Weight status (%)^c^			
Thin	1.2	0.8	
Normal weight	73.2	74.6	
Overweight	17.1	17.8	
Obese	8.5	6.8	<0.01
FAS^a^,^b^	6.5±1.8	6.7±1.6	0.70

FAS, Family Affluence Scale.

a Values are mean±standard deviation.

b Between-sex analysis by Mann–Whitney U test.

c Analysis by *χ*^2^ for categorical variables.

Results from urine collection are shown in Table 2. Boys showed a greater median sodium and potassium excretion than girls (*p<*0.01). Of all participants, 83% exhibited sodium intake above upper-limit recommendations (2,000 mg/day), while 96.1% showed potassium intake below recommendations (3,510 mg/day). The Na^+^/K^+^ ratio were similar in both sexes (1.7 in boys and 1.6 in girls, *p*=0.11). The coefficient of variation was 43.2% for sodium excretion, 32.3% for potassium excretion, and 26.8% for creatinine excretion.

**Table 2:** Urinary data on sodium and potassium excretion by sex (13–18 years)^a^

	Boys *n*=82	Girls *n*=118	*p*
Sodium (mg/day)^a^,^b^	3,725±1,445	3,062±1,379	<0.01
% of sodium excretion below 2,000 mg/day^c^	9.8	22	
Potassium (mg/day)^a^,^b^	2,237±704	1,904±593	<0.01
% of potassium excretion above 3,510 mg/day^d^	6.1	1.7	
Na/K ratio^a^,^b^	1.7±0.6	1.6±0.6	0.11

Na/K ratio, sodium-to-potassium ratio.

a Values are mean± standard deviation.

b Analysis by Mann–Whitney U test.

c According to the WHO guidelines for sodium intake (15).

d According to the WHO guidelines for potassium intake (16).

In this study, sodium and potassium intakes determined by dietary records correlated (weak correlations) with 24-h sodium and potassium excretion values (*r*=0.152. *p*=0.03 for sodium and *r*=0.229, *p<*0.01 for potassium).

As shown in Table 3, boys reported higher EI than girls (*p<*0.01). From dietary records, mean sodium intake was 2,649 mg/day for boys and 2,106 mg/day for girls (*p<*0.01), which corresponds to a mean estimated salt intake of 7 g/day for boys and 5 g/day for girls. Mean potassium intake was 2,998 mg/day for boys and 2,471 mg/day for girls (*p*=0.01).

**Table 3:** Dietary intake of the sample by sex (13–18 years) crude and adjusted to total energy intake (*n* = 178)

	Boys	Girls	*p*	p*
Nutritional intake				
Total energy (kcal/day)^a^,^b^	2,449±929	1,981±665	<0.01	<0.01
Carbohydrates (g/day)^a^,^b^	285±113	242±83	0.01	0.31
Carbohydrates (%TEI)^a^,^b^	47±7	49±8	0.08	0.20
Sugars (g/day)^a^,^b^	131±64	115±57	0.02	0.41
Protein (g/day)^a^,^b^	103±53	79±32	<0.01	0.61
Protein (%TEI)^a^,^b^	17±4	16±4	0.53	0.38
Fat (g/day)^a^,^b^	93±42	73±34	<0.01	0.91
Fat (% TEI)^a^,^c^	34±7	33±7	0.21	0.81
Dietary fiber (g/1,000 kcal)^a^,^b^	6±3	7±3	0.07	0.09
Total food (g/day)^a^,^b^	2,540±916	2,219±735	0.02	0.89
Total water (g/day)^a^,^b^,^d^	1,781±713	1,632±609	0.22	0.46
Potassium (mg/day)^a^,^b^	2,998±1,368	2,471±1,068	0.01	0.52
Sodium (mg/day)^a^,^b^,^e^	2,649±1,586	2,106±1,204	<0.01	0.29
Salt (g/day)^a^,^b^,^e^	7±4	5±3	<0.01	0.29
Na/K ratio^a^,^b^	1.7±0.6	1.6±0.6	0.19	0.35
Dietary intake				
Cereal and cereal products (g/day)^b^,^f^	286 (227, 357)	225 (162, 309)	0.01	0.22
Breads (g/day)^b^,^f^	93 (45, 143)	80 (45, 120)	0.26	0.67
Breakfast cereals (g/day)^b^,^f^	30 (30, 40)	30 (30, 40)	0.12	0.52
Biscuits, cakes, puddings, scones, doughnuts (g/day)^b^,^f^	60 (29, 124)	75 (40, 117)	0.52	0.05
Pasta, rice, and other cereal-based products (g/day)^b^,^f^	151 (97, 224)	97 (43, 180)	<0.01	0.01
Meat products (g/day)^b^,^f^	160 (120, 252)	132 (80, 212)	0.01	0.80
Chicken and turkey dishes (g/day)^b^,^f^	118 (93, 220)	105 (99, 173)	0.44	0.87
Sausages (g/day)^b^,^f^	40 (20, 60)	25 (14, 45)	0.12	0.17
Bacon and ham (g/day)^b^,^f^	30 (23, 60)	30 (23, 45)	0.43	0.83
Red meats (g/day)^b^,^f^	119 (90, 195)	112 (56, 160)	0.10	0.33
Milk and milk products (g/day)^b^,^f^	545 (292, 841)	471 (304, 659)	0.07	0.17
Milk (g/day)^b^,^f^	516 (283, 841)	400 (280, 545)	0.01	0.01
Cheeses (g/day)^b^,^f^	30 (28, 60)	30 (28, 56)	0.86	0.71
Vegetable and potato products (g/day)^b^,^f^	123 (73, 175)	121 (63, 224)	0.84	0.18
Potato products (g/day)^b^,^f^	155 (93, 350)	155 (116, 231)	0.77	0.86
Soups and sauces (g/day)^b^,^f^	363 (117, 541)	363 (174, 368)	0.50	0.71
Vegetable soups (g/day)^b^,^f^	363 (363, 541)	363.0 (261.5, 458.8)	0.10	0.18
Sauces (g/day)^b^,^f^	15 (11, 68)	10 (8, 20)	0.18	0.32
Fruits (g/day)^b^,^f^	301 (174, 487)	174 (132, 328)	0.10	0.19
Fish and seafood dishes (g/day)^b^,^f^	184 (108, 272)	120 (75, 227)	0.14	0.24
Cod fish (g/day)^b^,^f^	236 (71.0, -)	114 (68, 233)	0.50	0.55
Oils and fats (g/day)^b^,^f^	10 (4, 27)	10 (5, 21)	0.53	0.18
Egg and egg dishes (g/day)^b^,^f^	100 (58, 100)	46 (22, 50)	0.03	0.03
Sugars, preserves, and confectionery (g/day)^b^,^f^	16 (10, 37)	12 (8, 25)	0.07	0.92
Fast food (g/day)^b^,^f^	150 (100, 333)	150 (82, 202)	0.85	0.37
Pizza (g/day)^b^,^f^	220 (191, 445)	301 (200, 403)	0.94	0.29
Sandwiches, burgers, filled wraps (g/day)^b^,^f^	219 (219, 219)	219 (80, 219)	0.40	0.75
Salted snacks and fried snacks (g/day)^b^,^f^	104 (52, 229)	144 (88, 147)	0.77	0.46
Fried potatoes (g/day)^b^,^f^	100 (100, 150)	100 (50, 123)	0.21	0.26
Beverages (g/day)^b^,^f^	720 (476, 1,140)	825 (540, 1,047)	0.58	0.04
Soft drinks (g/day)^b^,^f^	435 (200, 615)	495 (220, 707)	0.37	0.11
Water (g/day)^b^,^f^	410 (90, 720)	440 (140, 846)	0.54	0.73
Hot beverages (g/day)^b^,^f^	30 (4, 122)	102 (10, 295)	0.20	0.34
Other foods (g/day)^b^,^f^	69 (22, 185)	81 (21, 127)	0.83	0.93

TEI, Total Energy Intake.

a Values are mean±standard deviation.

b Analysis by Mann–Whitney U test.

c Analysis by Student's *t*-test for continuous variables.

d Water from foods, beverages, and metabolic water.

e Estimated from dietary records without considering household salt, 393 mg sodium = 1 g salt.

f Values are median (P25, P75).

* Adjusted for energy intake.

**Fig. 1:**
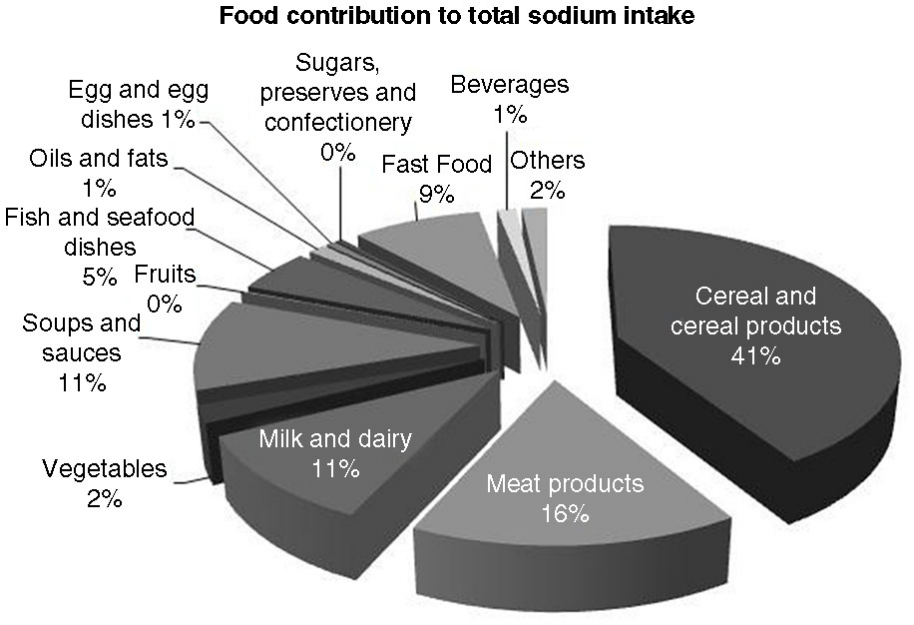
Contribution (%) of dietary sources for total sodium intake to total sample of subjects (*n* = 178). The contribution of each food category is expressed in mean percentage.

The major food sources of sodium in participants’ diet (Fig. 1) were cereal and cereal products (mean 41%: including bread, 16%; pasta, rice, and other cereal-based products, 12%; and breakfast cereals, 5%); meat products (mean 16%: including bacon and ham, 7%; sausages, 4%; red meats, 3%; and chicken and turkey dishes, 2%); and milk and milk products (mean 11%: including milk, 4%, and cheese, 4%). Fast food contributed a mean of 9% to total sodium ingestion, including the contribution of pizza (mean 3%), salted and fried snacks (mean 1%), fried potatoes (mean 3%) and sandwiches, and burgers and filled wraps (mean 2%).

**Fig. 2:**
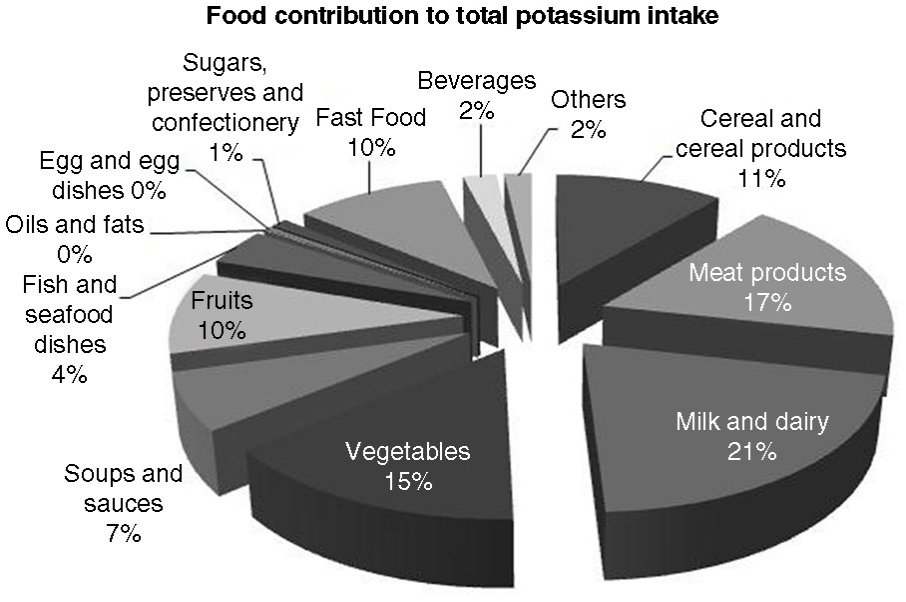
Contribution (%) of dietary sources for total potassium intake to total sample of subjects (*n* = 178). The contribution of each food category is expressed in mean percentage.

By contrast, milk and milk products (mean 21%: including milk, 14%, and cheese, 1%), meat products (mean 17%: including red meats, 9%; chicken and turkey dishes, 5%; bacon and ham, 2%; and sausages, 1%) and vegetables (mean 15%) were major sources of total potassium intake (Fig. 2). Fast food contributed 10% to total potassium ingestion, including the large contribution of fried potatoes (mean 8%).

The contribution of food groups to total sodium and potassium intake by sex, considering only participants who ingested foods included in the groups is shown in Table 4.

**Table 4:** Contribution (%) of dietary sources for total sodium and potassium intake by sex considering subjects that consume those food groups (*n* = 178)

	% contribution to sodium intake						% contribution to potassium intake					
		
	*n*	Boys	*n*	Girls	*p*	p*	*n*	Boys	*n*	Girls	*p*	p*
Cereal and cereal products	73	39.5±16.2	105	42.5±18.0	0.37	0.54	73	10.9±7.7	105	11.3±8.8	0.92	0.59
Breads	62	18.1±10.7	85	19.4±11.6	0.63	0.76	62	4.6±3.1	85	5.1±3.9	0.68	0.63
Breakfast cereals	36	9.0±5.9	47	10.6±7.6	0.26	0.87	36	3.4±3.6	47	4.1±3.0	0.03	0.67
Biscuits, cakes, puddings, scones, doughnuts	45	10.0±7.6	75	16.4±10.4	<0.01	<0.01	45	4.6±4.6	75	4.6±4.6	0.73	0.68
Pasta, rice, and other cereal-based products	60	16.5±11.7	83	14.1±10.8	0.13	0.11	60	3.3±3.7	83	3.4±5.5	0.07	0.23
Meat products	71	18.3±14.1	95	15.7±13.5	0.16	0.69	71	19.3±11.8	95	18.2±12.8	0.25	0.58
Chicken and turkey dishes	25	4.4±2.7	42	5.3±4.0	0.36	0.65	25	12.3±9.1	42	12.6±8.7	0.63	0.17
Sausages	22	17.9±12.7	14	16.3±12.4	0.63	0.82	22	3.6±2.6	14	3.0±1.9	0.77	0.73
Bacon and ham	31	15.1±9.9	42	18.2±8.2	0.06	0.12	31	5.2±4.0	42	5.4±4.6	0.75	0.79
Red meats	48	6.9±8.4	54	4.8±6.9	0.09	0.30	48	16.9±11.3	54	15.9±11.5	0.63	0.28
Milk and milk products	71	10.4±7.8	97	12.0±8.9	0.31	0.38	71	21.6±13.0	97	22.7±13.4	0.85	0.63
Milk	61	5.2±5.0	85	5.2±4.2	0.37	0.73	61	17.6±10.7	85	16.9±11.7	0.47	0.15
Cheeses	26	10.5±6.5	35	12.4±7.4	0.23	0.35	26	3.1±2.3	35	3.2±2.8	0.92	0.78
Vegetable and potato products	45	5.3±11.6	77	1.7±3.9	0.93	0.63	45	16.1±16.8	77	23.3±19.2	0.16	0.13
Potato products	24	8.8±15.2	47	2.0±4.6	0.11	0.33	24	27.3±16.8	47	31.5±17.8	0.36	0.98
Soups and sauces	34	23.0±15.2	42	28.6±15.1	0.15	0.96	34	16.3±11.9	42	15.2±10.8	0.89	0.52
Vegetable soups	24	29.6±12.0	32	31.4±12.9	0.70	0.29	24	21.3±9.6	32	19.2±9.1	0.39	0.10
Sauces	9	4.8±5.3	7	2.3±1.1	0.96	0.87	9	2.9±3.6	7	1.3±2.2	0.79	0.63
Fruits	36	0.2±0.4	61	0.1±0.1	0.31	0.02	36	17.9±11.7	61	17.7±15.3	0.64	0.12
Fish and seafood dishes	18	17.0±17.6	36	17.2±15.3	0.74	0.75	18	17.1±14.5	36	12.9±11.8	0.36	0.12
Cod fish	3	22.9±13.9	13	24.3±19.1	0.84	0.84	3	1.6±1.1	13	2.4±2.5	0.64	0.74
Oils and fats	34	2.2±2.9	61	2.2±3.8	0.91	0.61	34	0.1±0.1	61	0.1±0.2	0.48	0.83
Egg and egg dishes	5	6.8±5.4	17	4.0±3.3	0.29	0.78	5	3.3±1.4	17	3.1±2.3	0.72	0.29
Sugars, preserves, and confectionery	42	1.3±2.1	59	0.8±1.0	0.57	0.43	42	2.6±6.2	59	1.4±2.0	0.73	0.94
Fast food	38	18.5±17.3	35	24.0±20.1	0.28	0.11	38	25.9±15.3	35	26.7±14.9	0.83	0.92
Pizza	5	36.6±19.8	8	41.3±13.0	0.56	0.72	5	23.7±19.3	8	19.9±8.9	0.59	0.81
Sandwiches, burgers, filled wraps	7	27.0±16.6	7	22.2±19.7	0.75	0.41	7	15.9±13.0	7	13.6±6.0	0.66	0.57
Salted snacks and fried snacks	4	21.2±11.2	4	27.1±19.4	0.56	0.99	4	10.5±3.0	4	7.7±6.5	0.51	0.99
Fried potatoes	32	7.7±4.7	26	9.4±8.1	0.73	0.78	32	23.0±10.1	26	24.8±13.2	0.90	0.55
Beverages	71	1.2±1.1	105	1.4±1.2	0.09	0.08	71	2.6±7.4	105	2.4±5.3	0.02	<0.01
Soft drinks	45	0.9±1.2	53	1.2±1.5	0.20	0.20	45	0.3±0.3	53	1.4±3.9	0.84	0.49
Water	55	0.7±0.6	85	0.9±0.7	0.19	0.41	55	0.6±1.6	85	0.5±1.0	0.36	0.05
Hot beverages	9	0.6±0.8	26	0.2±0.2	0.76	0.49	9	14.9±15.7	26	5.2±7.4	0.23	0.38
Other foods	16	6.7±8.2	28	7.2±9.3	0.63	0.53	16	8.3±11.0	28	7.0±10.1	0.48	0.76

The contribution of each food category is expressed in mean percentage±standard deviation.

* Adjusted for energy intake.

## Discussion

To the best of our knowledge, this study has marked the first in Portugal to estimate sodium and potassium intake in a large group of adolescents aged 13–18 years using 24-h urine excretion. Our results have shown that 83% of participants exceed the recommended sodium intake and that 96.1% did not meet recommendations for potassium intake.

Portuguese data related to sodium excretion are scarce. A study of Portuguese children aged 10–12 years showed that sodium excretion was 3,072±985 mg/day (35), while a more recent study with children aged 8–10 years reported that median sodium excretion was 2,737 mg/day in boys and 2,104 mg/day in girls (36). Comparing our results to those representing other European adolescents, Portuguese adolescent boys seem to have a mean sodium excretion greater than that of Italian (2,967 mg/day) (37), Spanish (3,270.6 mg/day) (38), English (i.e. London) (3,401.47 mg/day) (25), and German (3,013 mg/day) adolescents (39). Similar results were found between Portuguese and other European adolescent girls, except the Spanish (2,888.8 mg/day) (38). Across the board, sodium excretion was greater in boys than in girls (25, 36–39), probably due to their higher food intake.

Conversely, other studies have evaluated sodium intake in children and adolescents via dietary recall. In Australian adolescents (aged 14–16 years), mean dietary sodium intake was 3,190 mg/day, a value that increased with age (40). In French adolescents, median sodium intake was 2,245 mg/day (41) and in Korean adolescents mean sodium ingestion was 4,100 mg/day (42). Results from our study related to sodium intake via dietary recall are more similar to French adolescents, which is lower than Korean and Australian adolescents.

The amount of potassium excretion in our study was well below the minimum value recommended by the WHO, but it is comparable with those reported by Geleijnse et al. (43) regarding 233 Dutch children aged 5–17 years, among whom mean potassium excretion was 1,704.3 mg/day and was higher in boys. Among Portuguese children (8–10 years old), mean potassium excretion was 1,701±594 mg/day in boys and 1,682±541 mg/day in girls (36).

Na^+^/K^+^ ratio intake is a critical risk factor for hypertension (44), and in our study, mean urinary Na^+^/K^+^ ratio was 1.7 in boys and 1.6 in girls; these values are higher than Na^+^/K^+^ <0.59 mg/mg the WHO recommends (45). Higher Na^+^/K^+^ ratio is associated with Western diets heavily reliant on processed foods, high in added sodium, and low in potassium (38).

Na^+^/K^+^ ratio excretion among our study's participants was smaller than the median ratio found in Portuguese children (2.73 in boys and 2.33 in girls) (36) and in Dutch adolescents (ratio was 3.3) (43), as well as in Italian children and adolescents (ratio was 3.79±1.68) (46). However, the ratio in our participants is still three times greater than 0.59 mg/mg, which raises concerns about poor dietary behavior early in childhood.

Participants in our study did not meet the recommended intake for sodium or potassium, and major dietary contributors of sodium and potassium were identified in order to improve food-based intervention programs.

Correlations between dietary and urinary data found in our study were comparable with those reported by other authors (41, 47). The possible explanation of this result could be the difficulty in maintaining food records that accurately estimate food quantities and food ingredients, especially if the subjects eat out of home.

Taking into account the overall intake reported for our sample of adolescents, the food groups that contribute most to total sodium intake are cereals and cereal products (mean 41%) and meat products (mean 16%). Meaning that, in addition to bread (mean 16%), sodium added during the cooking process is the chief source of sodium intake as in pasta, rice, and other cereal-based products (mean 12%), meat products (mean 16%), fish and seafood products (mean 5%), and vegetable soup (mean 10%). Our data show that in individuals who consumed vegetable soup (approximately 15% of participants), soup contributed roughly a third to their total sodium intake (29.6% for boys and 31.4% for girls in relation to total dietary sodium intake; Table 4). Conversely, in individuals who consumed processed foods such as fast food (about 41% of participants), such foods became an important contributor to their total sodium intake.

Since the largest proportion of sodium is added during food manufacture or preparation, including for vegetable soup and bread, changes in recipes to reduce sodium content could be possible without disturbing other nutrient values (48) and consumer preferences. In a study of children and the elderly, 30% salt reduction in vegetable soup was achieved without compromising perceived saltiness (49). This result is especially important in Portugal where the amount of sodium in a 300 g portion of vegetable soup cooked outside home may be as high as 1,316 mg (50).

To diminish salt added during cooking processes, it is necessary to devise nutrition education strategies that increase public awareness of salt reduction and develop technology to control the amounts of added salt.

At the same time, industry and policy makers need to engage efforts to make processed foods with low sodium content available on the market. Portugal's salt reduction plan (51) includes a strategic objective to increase the availability of products with low salt content, via the participation of the industry. Therefore, since 2009 in Portugal, sodium content in bread was legally limited to 550 mg/100 g (52), although this limit was exceptionally permissive compared with that of other countries’ legislation (53, 54). Bread is probably the most important staple food of modern Portuguese diet (55) as well as of the traditional Mediterranean diet of the early 1960s (56). Although salt (i.e. sodium chloride) has a technical and functional role in the manufacture of bread (57), the production of low-salt bread is feasible (57), and incremental reductions of 30 to 50% have proven to be acceptable to consumers (58–60).

Surprisingly, the top sources of potassium among all sample of participants were milk and milk products (21%) and meat products (17%), as explained by the low intake of fruit and vegetables (2 and 9%, respectively; data not shown). These data may reflect the low proportion of adolescents who follow the recommendation to eat at least 400 g of fruit and vegetables daily (61). The intake of fruits and vegetables as a natural source of potassium is inversely related to the risk of stroke (62) and is associated with more varied and higher quality diet (63).

Food processing reduces the natural amount of potassium in many food products (64). Adolescent diets in our study were high in processed foods and low in fresh fruits and vegetables with high sodium intake. It suggests that Portuguese adolescents have moved away from a typical Mediterranean diet toward the dietary patterns common in industrialized countries (41). Developing strategies to improve fruit and vegetable consumption would thus increase potassium intake, as well as offer other beneficial health effects (65).

In the context of our study, vegetable soup deserves particular attention, for it is a staple food of the Mediterranean diet and of Portuguese dietary habits, consumed daily by 67% of population (66) and promoted by healthy eating education programs (67). Portuguese-style soups are rich in vegetables, low in energy density, and high in dietary fiber, all of which are associated with a lower risk of obesity (68). In our study, vegetable soup was an important contributor to total potassium intake; however, its contribution to total sodium was exceptionally high and therefore detrimental. This typical food seems to show the potential for improving potassium intake if its consumption could be stimulated in adolescents and added salt were diminished.

A strategy that emphasizes reducing sodium added to foods, promotes potassium-rich foods, and promotes reformulation of processed and packaged foods could achieve greater public health benefits than restricting sodium alone (69).

Our study is strengthened by the 24-h urine collection, the clinical gold standard method, and by our sample size (considering that a representative sample of 120 to 240 individuals from each population subgroup is needed to estimate population-level sodium intake according to WHO (21)). The 24-h urine collection objectively measured dietary sodium intake from food and from salt added during cooking and at the table. Bias stemming from under collection or overcollection was decreased by 24-h urine creatinine excretion quality control and self-reported total daily collection. The fact that we performed only one urine collection per participant could be a limitation; however, urine collections were measured in a large quantity of participants and showed a response rate of 63% similar to that of other studies (19).

Another potential limitation of our study is the possible underestimation of sodium and potassium intake in 24-h dietary recall, as has been previously reported (47, 70). Nevertheless, 24-h recalls were administered by trained researchers using a photographic book and household measures to quantify portion sizes, which minimized this potential limitation. It should also be noted that the food composition data in software used for calculating nutrient intakes might also introduce bias into dietary data. To avoid this, we carefully checked the sodium and potassium composition in the foods consumed by participants. We should additionally note that the collection of urine and the respective diet recall refers to Sunday. Although variation in day-to-day dietary intake exists, at their age, our participants were more likely concerned about what their peers think about them, and the thought of completing urine collection during weekdays was roundly rejected by adolescents and their parents at the outset of the study. For this reason, we performed urine data collection on Sunday only.

## Conclusions

We found that Portuguese adolescents have high sodium intake and low potassium intake compared with WHO recommendations. The low potassium intake raises concerns about the potential detachment of Portuguese adolescents’ dietary habits from the traditional Mediterranean diet with increased intake of processed foods and few vegetables. Sodium reduction in widely consumed staple foods such as cereal products/bread and vegetable soup could be effective for decreasing sodium intake, while the promotion of milk, fruit, and vegetable intake could similarly increase potassium intake.
